# Editorial: Genetic and Epigenetic Control of Immune Responses

**DOI:** 10.3389/fimmu.2021.775101

**Published:** 2021-10-05

**Authors:** Satish kumar R. Noonepalle, Lidia Karabon, Katherine B. Chiappinelli, Alejandro Villagra

**Affiliations:** ^1^ Department of Biochemistry and Molecular Medicine, GW Cancer Center, School of Medicine and Health Sciences, George Washington University, Washington DC, United States; ^2^ Department of Experimental Therapy, Ludwik Hirszfeld Institute of Immunology and Experimental Therapy, Polish Academy of Sciences, Wroclaw, Poland; ^3^ Department of Microbiology, Immunology, and Tropical Medicine, GW Cancer Center, School of Medicine and Health Sciences, George Washington University, Washington DC, United States

**Keywords:** tumor microenvironment, tumor immunology, tumor immune escape mechanisms, checkpoint inhibition therapy, genetic and epigenetic alterations, DNA methylation, HDAC inhibitor

## Introduction

**Figure 1:**
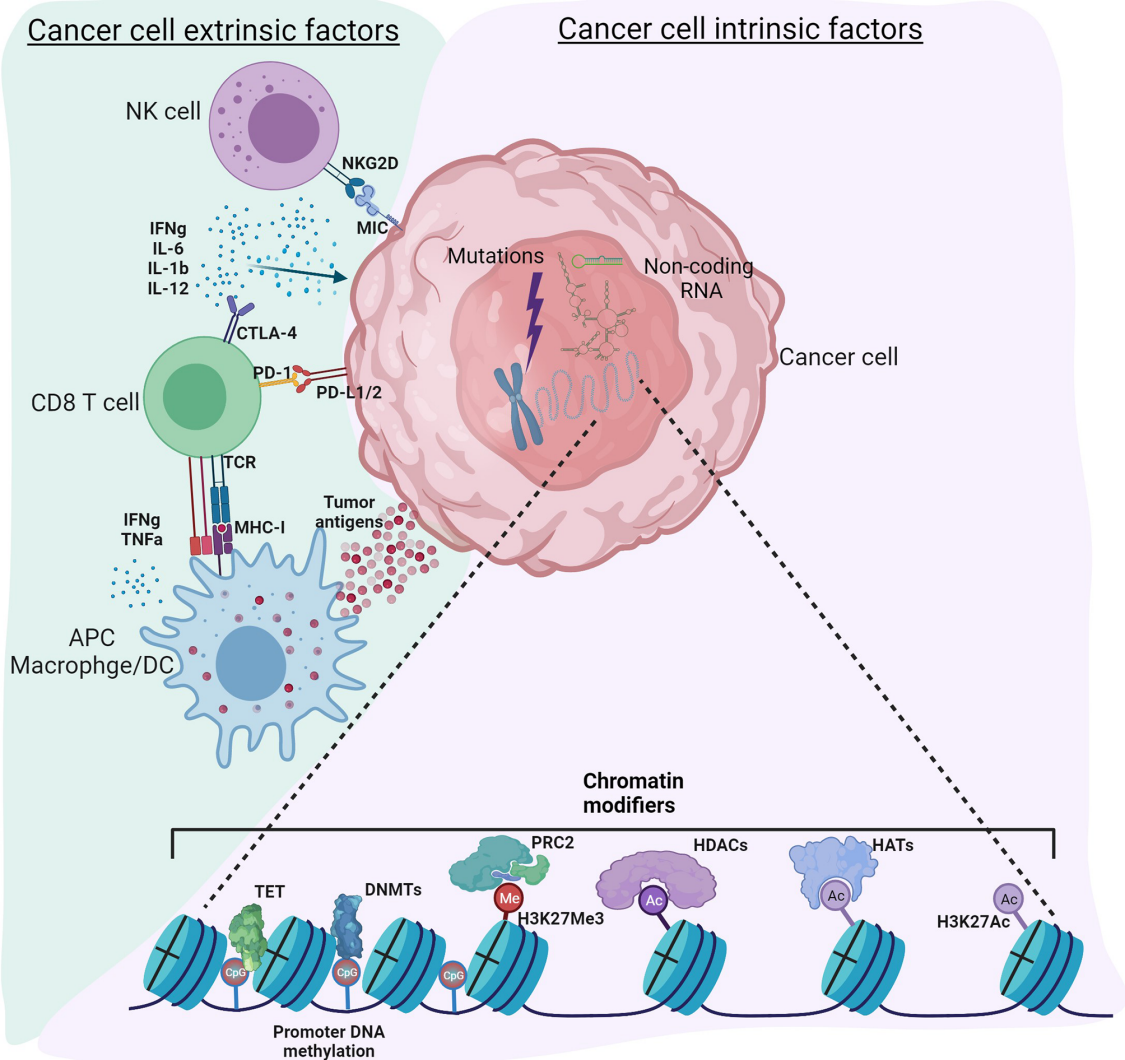
Tumor intrinsic and extrinsic factors regulating immune responses in the tumor microenvironment. Cancer cell intrinsic factors such as genomic mutations, chromatin modifiers and non-coding RNA regulate tumor initiation, propagation as well as immunogenicity. Epigenetic modifications such as DNA methylation, histone acetylation and methylation regulate gene expression. Non-coding RNA including long non-coding RNA, microRNA, circular non-coding RNA regulate gene transcription as well as mRNA stability. Other mechanisms intrinsic to cancer cells include expression of immunosuppressive cytokines to facilitate escape from anti-tumor immunity, expression of immunosuppressive molecules such as PD-L1 and PD-L2, suppression of antigen processing and presentation machinery and tumor associated antigens. Cancer cell extrinsic factors include tumor infiltrated immune cells, fibroblasts, stromal cells and endothelial cells. Extrinsic factors also include secretory factors such as cytokines, chemokines, metabolites, growth factors and immune checkpoint molecules. Tumor associated antigens presented by antigen presenting cells such as macrophages, dendritic cells activate CD8 T-cells for effective anti-tumor immunity. However, immune checkpoint molecules expressed by cancer cells regulate the inflammatory status of the tumor tamping down the inflammation. Use of epigenetic modifiers such as DNMT inhibitors, HDAC inhibitors, BET inhibitors etc. can alter these reversible modifications to enhance anti-tumor immunity. Figure created with BioRender.com.

Cancer, traditionally viewed as a disease driven by genomic alterations, is now perceived as an accumulation of genetic mutations as well as global epigenetic changes to the chromatin that regulate gene expression (1, 2). Genetic alterations to either tumor suppressors or oncogenes can result in dramatic gene expression changes leading to cancer; however, changes in the epigenome are rather subtle. Despite similar genomic sequences in all the cell types, the epigenome can vary considerably, resulting in distinct gene expression patterns and, therefore, distinct cellular functions. Epigenetic modifications to chromatin include DNA methylation, histone modifications, nucleosome positioning, and non-coding RNAs that can regulate access of DNA to transcription factors and other cis-regulatory elements, thereby affecting gene expression (3). It is now recognized that genetic and epigenetic components complement each other to drive tumor initiation and progression (4). Recent technical advances in high throughput sequencing have improved understanding of the epigenomic landscape at a higher resolution. Massive datasets and databases, including the encyclopedia of DNA elements (ENCODE) (5), The Cancer Genome Atlas (TCGA) ((https://www.cancer.gov/tcga), the NIH Roadmap Epigenomics Mapping Consortium (6), the Epigenome browser (7), have enhanced our ability to understand the interplay between cancer cells, tumor microenvironment (TME) and immune cells. Therefore, a new classification of molecular epigenetic modifications is needed to differentiate “cancer intrinsic” and “cancer extrinsic” mechanisms influencing anti-tumor immune responses. The schematic shows a graphical representation of various intrinsic and extrinsic factors affecting the cancer cells and thereby regulate the TME (**Figure 1**). The International Human Epigenome Consortium (IHEC) provides high-resolution reference epigenomes of major primary human cell types (8). Based on the data from these projects, genetic and epigenetic crosstalk in cells is evident, and it has led to the identification of novel biomarkers and the development of novel therapeutic strategies. The articles in this Research Topic on Genetic and Epigenetic Control of Immune Responses address both cell-intrinsic and cell-extrinsic mechanisms controlling the immune response to tumors.

Several articles utilized publicly available databases to investigate the relationship between tumor and immune cells in the microenvironment and, consequently, response to immune therapies. In this Research Topic, Zhuang et al. investigated the relationship between immune-related genes and the outcome of lung squamous cell carcinoma (LUSC) using datasets from TCGA and defined an immune gene risk index. The immune gene risk index served as an independent prognostic factor indicating that infiltration of neutrophils and dendritic cells was strongly associated with the high-risk group. Another prognostic model in this Research Topic designed by Xu J et al., with machine learning, indicated that LYN, C3, COPG2IT1, LA.DQA1 and NFRSF17 may serve as novel biomarkers to assess the prognosis of patients with non-small cell lung cancer and ICB therapy. Systematic analysis of skin cutaneous melanoma data from TCGA by Qu et al. indicated that ETS family member ETV7 transcription factor expression was downregulated in melanoma tumors and associated with poor prognosis. Further gene enrichment analysis and immune profile analysis indicated that ETV7 regulates differentiation and activation of T cells (Qu et al.).

Regarding immune-related features, the triggering receptor expressed on myeloid cells 2 (TREM2), a transmembrane receptor of the immunoglobulin superfamily, played an important role in tumor progression by modulating immune responses of TAMs in the TME (9). Meta-analysis of TREM2 by Cheng et al., across 33 different cancer types from various databases, including TCGA, indicated that TREM2 gene expression negatively correlated with the level of infiltration of most immune cells but positively correlated with infiltration levels of M1 and M2 macrophages in 6 different cancer types. Integrative pan-can analysis of cancer datasets by Xu W-X et al. concluded that Lipocalin 2 (LCN2), a novel innate immune protein, is a potential biomarker for immune infiltration and poor prognosis in cancers. Analysis of TCGA datasets for Hepatitis B virus-related hepatocellular carcinoma also indicated a significant difference in prognosis based on immune phenotypes associated with higher expression of metabolic and stem cell-related genes (Zhang et al.). Based on the aforementioned research studies in this Research Topic, it can be concluded that cancer genomic and transcriptomic databases have revolutionized the computational approach to understanding the tumor microenvironment.

Due to the reversible nature of epigenetic changes, epigenomic modulators as therapeutic agents are gaining more attention to influence the TME towards antitumor immunity. In this editorial, we will highlight the latest studies on genetic and epigenetic factors that influence the fate of cancer cells and immune cells as well as the factors that shape the TME. Finally, we will discuss unique epigenetic profiles of cancer cells and immune cells with exciting possibilities that link the TME and changes in the gene expression profiles of the immune cells. Finally, we will explore the possibility of using epigenetic modifiers as drug targets either alone or in combination with immunotherapies.

## Tumor Immunology and Cancer Immunotherapy

The immune system plays a major role in both the eradication and the establishment of tumors. Active immune surveillance by the innate immune system can identify and eliminate nascent tumor cells, eradicating cancer (10). However, the establishment of cancer indicates that tumor cells successfully evade host immune defenses through a process called immunoediting, which is divided into three phases: elimination, equilibrium, and escape (11). An early study indicated that IFNγ producing lymphocytes prevented tumorigenesis in mice with an intact immune system. However, tumors that developed by escaping immune detection were less immunogenic than those developed in immunodeficient mice, supporting the concept of cancer immunoediting (12). Recent advances in immunotherapy have revolutionized cancer therapies. The basic premise of cancer immunotherapies is to potentiate the ability of T-cells to recognize and eliminate cancer cells. Based on the infiltration of immune cells, tumors can be classified as hot or cold tumors. Tumors with poorly infiltrated immune cells are often called “cold tumors” whereas tumors with inflammation resulting from heavy infiltration of immune cells are called “hot tumors” (13). Patients with so-called cold tumors are non-responsive to immunotherapy resulting in primary resistance.

On the other hand, patients exhibiting an initial response to immunotherapy can eventually acquire resistance due to immunoediting (1). The differentiation of naïve T-cells into anti-tumor effector T-cells, as shown in the schematic, begins with the formation of an immune synapse between the T-cell receptor (TCR) and the antigenic peptide presented by major histocompatibility complex (MHC) of antigen-presenting cells (APCs), which serves as signal 1 (14). Signal 2 involves binding co-stimulatory molecules like the interaction between CD28 and B7 molecules for complete T-cell activation. Lack of signal 2 despite effective antigen presentation leads to T-cell anergy (15). This step is tightly regulated by countering immune checkpoint molecules to prevent prolonged activation and autoimmunity (16). Finally, signal 3 involves cytokine stimulation that facilitates proliferation and clonal expansion of effector T-cells (17).

The impetus for cancer immunotherapy was derived from ipilimumab’s phase 3 clinical study, which blocks cytotoxic T-lymphocyte-associated antigen 4 (CTLA-4), an immune checkpoint molecule, to potentiate T-cell mediated anti-tumor response. Anti-CTLA-4 treatment improved overall survival in a large cohort of metastatic melanoma patients (18). CTLA-4, due to its high affinity for B7 ligands, competes with CD28 and inhibits T-cell proliferation and IL-2 secretion (19). Another immune checkpoint molecule expressed by reactive T-cells is programmed death 1 (PD-1), and its ligands PD-L1 and PD-L2 expressed by tumors cells and other immune cells when engaged result in T cell dysfunction (20). The subsequent discovery of other immune checkpoint molecules such as lymphocyte activation gene-3 (LAG-3), T cell immunoglobulin and mucin-domain containing-3 (TIM-3), T cell immunoglobulin and ITIM domain (TIGIT), and V-domain Ig suppressor of T cell activation (VISTA) has led to the exploration of ICB therapy for numerous cancer types (21).

Epigenetic modifications play a critical role in regulating the expression of immune checkpoint molecules. DNA methylation (5-methylcytosine) is an epigenetic silencing mark associated with hemimethylated CpG palindrome sequences (approximately 1 kb CpG-rich regions) known as CpG islands (22). Analysis of breast tumor tissue and normal breast tissue for the expression of immune checkpoint molecules indicated that CpG islands in the promoter regions of PD-1, CTLA-4, and TIM-3 were hypomethylated (loss of DNA methylation) in tumor tissues compared with normal tissues. CpG islands of PD-L1 and LAG-3 were hypomethylated, whereas TIGIT was hypermethylated in both normal and breast tumor tissues. This methylation data was inversely correlated with gene expression. H3K9me3 and H3K27me3 marks were reduced in the promoter loci of PD-1, CTLA-4, TIM-3, and LAG-3 in breast tumor tissues suggesting that epigenetic mechanisms affecting the cancer cell side of the equation can affect immune cells and vice versa (23). In the presented Research Topic Wagner et al. further provided an extensive analysis of immune checkpoint molecules and inherited variations as a marker for cancer risk and indicate that the variants in genes encoding these molecules might be considered as low-risk variants (OR<2) for cancer development, which has been well documented by numerous reports for *CTLA-4, PDCD1, PD-L1* genes, while still more studies are needed for *BTLA, TIM3, LAG3* and *TIGIT*.

## Cancer Intrinsic Epigenetic Mechanisms

As described by Hanahan and Weinberg, Hallmarks of cancer comprise six cellular processes that include sustaining proliferative signaling, resisting cell death, enabling replicative immortality, evading growth suppressors, inducing angiogenesis, and activation of invasion and metastasis (1). Underlying genomic instability further expedites the ability of cancer cells to attain these hallmarks. Epigenomic deregulation adds another layer of complexity to tumorigenesis (24); for example, genome-wide DNA hypomethylation can induce genomic instability (25). Stage IV metastatic colon cancer patients often have tumors with defective DNA mismatch repair and high microsatellite instability (MSI-High) (26). In this Research Topic Lin et al. report that analysis of gene expression and mutational data of colon and rectum adenocarcinomas from TCGA reveals that MSI-High tumors had higher infiltration of immune cell, expression of immune-related genes, and better immunogenicity than MSI-Low or microsatellite stable tumors. Therefore, patients with MSI-High colorectal cancer having MSI-High respond better to ICB therapy (27). Chromosomal instability is mediated by the loss of telomeres which protect the ends of chromosomes and prevent chromosome fusions (28). The expression of telomerase reverse transcriptase (TERT), a catalytic subunit of telomerase, regulated by multiple genetic and epigenetic alterations that affect tumors’ telomerase activity is presented as well in this Research Topic (Dratwa et al.). Therefore, tumor cell intrinsic genomic features mentioned above can significantly influence the initiation and propagation of cancer cells.

In this issue, a comprehensive analysis of the mutational status of tumor suppressor genes that include *TP53*, *CDKN2A*, *PTEN*, *RB1*, *BRCA1*, *BRCA2*, and immune-related gene expression in lung squamous cell carcinoma and lung adenocarcinoma samples from the TCGA database indicated that infiltration of immune cells was suppressed by tumors harboring mutations to the tumor suppressor genes (Kim et al.). This underscores the impact of the mutational status of tumor cells in shaping the TME and potentially dictating the usage of immunotherapeutic strategies in patients with mutations of tumor suppressor genes. Another study in this Research Topic by Wu et al. identified a prognostic TP53 associated immune signature in muscle-invasive bladder cancer based on differentially expressed immune-related genes between patients with or without TP53 mutations (29). The TP53 associated immune signature identified a high-risk group of patients characterized by increased infiltration of immunosuppressive regulatory T cells, myeloid-derived suppressor cells, and tumor-associated macrophages. This high-risk group of patients also had higher expression of CTLA4, LAG3, PDCD1, TIGIT, and HAVCR2, suggesting that they were more likely to respond to anti-PD-1 and neoadjuvant chemotherapy (Wu et al.).

A global survey of tyrosine kinase signaling identified c-ros oncogene 1 (*ROS1*) chromosomal rearrangements non-small-cell lung cancer (NSCLC) (30). ROS1 fusion NSCLCs are sensitive to crizotinib, a receptor tyrosine kinase MET inhibitor (31) but eventually develop resistance (32), and the effect of ROS1 fusion on ICB therapy is not known. In this Research Topic, Cai et al. reported that ROS1 fusion upregulated PD-L1 through activation of ROS1-SHP2 pathway using ROS1 fusion and crizotinib-resistant cell lines, suggesting that oncogenic driver mutations play a direct role in the expression of checkpoint PD-L1 molecule and facilitate the immune escape of NSCLC tumors.

Another mechanism of immune escape is downregulation of MHC-I molecules of antigen presentation machinery (33) through promoter hypermethylation (34), binding of polycomb repressive complex 2 (PRC2) at H3K27me3 repressive marks (35). Somatic mutations or loss of expression of the beta 2-microglobulin (B2M) gene in lung cancer cells can result in defective MHC class I expression, allowing the cancer cells to escape recognition by cytotoxic T cells (36). Due to genomic instability, mutated proteins expressed by tumor cells function as tumor-associated antigens (TAAs). In colorectal premalignant polyps, an estimated 11,000 genomic events per cell were detected (37). Most importantly, epigenetic driver mutations often dictate the success of immunotherapeutic approaches. For example, ARID1A driver mutations resulted in condensed chromatin of IFN responsive genes, reduced T-cell infiltration, and thereby anemic anti-tumor immunity (38). Epigenetic mechanisms in tumor cells also control the immune status of the TME as polycomb repressive complex 2 (PRC2) repressed the expression of Th-1 chemokines CXCL9 and CXCL10, resulting in poor T-cell infiltration in colon cancer (39), highlighting the complex interplay between tumor cells and immune cells in the TME. In the triple-negative breast cancer (TNBC) subtype with hypomethylated *IDO1* gene promoter compared to the estrogen receptor-positive breast cancer subtype with hypermethylated *IDO1* gene promoter resulted in the expression of IDO1 enzyme in the presence of activated CD8 T-cells, suggesting a counteractive mechanism employed by tumor cells to escape anti-tumor immunity (40, 41). Metabolic deregulation is often associated with epigenomic deregulation in cancer cells (42). Myeloproliferative neoplasms are not driven by BCR-ABL mutations in hematological malignancies, rather due to somatic mutations in JAK2 and exhibit metabolic vulnerabilities due to a high dependence on glucose metabolism. Here, Sharma et al. discuss the role of histone methylation and acetylation in metabolic deregulation of myeloproliferative neoplasms.

Moreover, in this Research Topic, increasing evidence indicates the role of non-coding RNA, including long non-coding RNAs (lncRNAs), in the immunomodulation of the TME (43). Hu et al. analyzed the gene expression data of patients with adenocarcinoma of esophagogastric junction from TCGA, identified 1470 differentially expressed lncRNAs, and narrowed them to an immune-related risk signature that can effectively predict the response to immunotherapy and chemotherapy. Li J-P et al. reported a seven lncRNA signature out of 331 immune-related genes (AC022784-1, NKILA, AC026355-1, AC068338-3, LINC01843, SYNPR-AS1, and AC123595-1) as a predictive model to forecast the progression of lung adenocarcinoma. Fang et al. discuss the regulatory roles of circular RNAs in the context of tumor immunology and immunotherapy.

## Cancer Extrinsic Epigenetic Mechanisms

Cancer extrinsic mechanisms constitute factors contributing to tumor initiation and progression not by the tumor cells but due to factors such as tumor-associated immune cells, stromal cells, and fibroblasts. These cells can directly influence epigenetic outcomes in tumor cells by secreting factors such as cytokines, chemokines, metabolites, growth factors, and other soluble factors. As shown in the schematic, we will discuss the role of predominant immune cells in the TME, including tumor-associated macrophages and infiltrated lymphocytes.

### Macrophages

Macrophages are a heterogeneous population of cells that play a critical role in enforcing both innate and adaptive arms of the immune system. Macrophages are terminally differentiated from circulating monocytes with their origin in the bone marrow, whereas evidence indicates that tissue macrophages progenitors are derived from the yolk sac and fetal liver during early embryonic hematopoiesis (44) with self-renewal capabilities (45). An extremely simplistic classification of macrophages based on the phenotypes is pro-inflammatory or classically activated (M1) macrophages and anti-inflammatory or alternatively activated (M2) macrophages (46). The M1/M2 nomenclature is originally linked to Th1/Th2 lymphocytes producing IFNγ or IL4 for activation of M1 or M2 macrophages, respectively. Macrophages are highly adaptable cells capable of responding to cues from the microenvironment and exhibit properties that make it difficult to strictly assign M1 or M2 phenotypes. Such plasticity of phenotypes demands remarkable changes in the epigenome resulting in distinct gene expression patterns (47). In the context of the TME, pro-inflammatory M1 macrophages are attributed to the anti-tumor activity, whereas anti-inflammatory M2 macrophages are deemed to be tumor-promoting. The complexity is even higher with tumor-associated macrophages (TAMs).

Macrophages differentiate from monocytes in the presence of colony-stimulating factors, resulting in significant gene expression changes (48). Epigenetic profiling of monocyte to macrophage differentiation uncovered approximately 8000 dynamic regions associated with at least 11000 DNase I hypersensitive sites suggesting a profound remodeling of chromatin (49). Differentiation was associated with demethylation catalyzed by ten-eleven translocation (TET) methylcytosine dioxygenases enzymes (50) of at least 114 Differentially methylated regions (DMRs) belonging to genes of the ERBB2, PDGFRβ, CXCR4, and PIK3 signaling pathways. Demethylated DMRs were also nucleosome depleted and enriched with activating histone marks H3K27ac and H3K4me1 in macrophages compared to monocytes (51). Exposure of macrophages to TLR ligands such as LPS and/or Th-1 cytokines such as IFNγ leads to M1 polarization by activating several epigenetic modifiers leading to transcription of pro-inflammatory cytokine genes mediated through the NF-κB (52), STAT1, HIF1α, IRF4 (53), and MAPK (54) pathways. Enhancer regions of loci that encode inflammatory genes are poised for gene expression with an open chromatin state marked by H3K4me1 and binding of macrophage lineage determining PU.1 and C/EBP transcription factors (55, 56). Upon activation signal, these cells readily express pro-inflammatory cytokines. Another epigenetic modifier, histone lysine methyltransferase EZH2, a member of the repressive PRC2 complex, increases H3K27me3 marks on the *SOCS3* gene leading to suppression of SOCS3 gene expression in activated M1 macrophages (57). Similarly, DNMT1-mediated hypermethylation of *SOCS1* resulted in decreased expression of SOCS1 and therefore increased expression of LPS-induced pro-inflammatory TNF and IL-6 cytokines in macrophages (58). Both SOCS1 and SOCS3 are negative regulators of the JAK/STAT pathway (59). On the contrary, repressive mechanisms mediated by negative histone marks H3K9me3, H3K27me3, H4K20me3, and repressors that bind inflammatory loci exist to prevent uncontrolled and chronic inflammation (60, 61). Jmjd3, an H3K27 demethylase deficiency, affected trimethylation of Irf4, a key transcription factor that regulated M2 macrophage polarization (62).

### Lymphocytes

IL-17 producing Th-17 cells, a subset of CD4 T-cells, are usually associated with proinflammatory function. Binding of Cxxc finger protein 1 (Cxxc1), a transcription factor with high affinity to unmethylated CpG sites at the *IL6R* gene promoter, retained H3K4me3 marks and regulated IL-6Rα expression, which mediates IL-6/STAT3 pathway and thereby controls the fate of CD4 differentiation towards T-regs or Th-17 CD4 T-cells (63). Lymphocytes isolated from HDAC11 knockout mice exhibited increased expression of *Eomes* and *Tbet* transcription factors and displayed enhanced proliferation, increased production of pro-inflammatory cytokines, and effector molecule expression suggesting that HDAC11 acts as a negative epigenetic regulator of T-cell effector function and phenotype (64). HDAC11 is shown to regulate the expression of OX40L and IL-10 producing T-regs in Hodgkin lymphoma (65). Entinostat, a synthetic benzamide-derived Class I HDAC inhibitor (66), enhanced NK cells’ ability to kill cancer cells by increasing the expression of MIC in tumor cells and NKG2D in primary human NK cells (67).

Inflammasomes are multiprotein complexes that sense danger signals emanated from cancer cells and mediate a pro-inflammatory response resulting in cell death through activation of cysteine proteases called caspases (68). Deregulation of inflammasome and chronic inflammation often damages healthy tissue along with tumor cells (69). Epigenetic mechanisms such as DNA methylation (70) and epigenetic readers such as BRD4 regulate NLRP3 gene expression (71). In this Research Topic, Zhong et al. reported that activation of NLRP3 inflammasome in an IL-1β dependent manner in AML cells promoted proliferation of leukemia cells by inhibiting apoptosis, resulting in resistance to chemotherapy. Another epigenetic mechanism to regulate inflammation described in this issue is mediated by microRNAs. MiR-124, which targets STAT3 and subsequently binding of STAT3 to the *IL17* gene promoter to activate it, in a Citrobacter rodentium infection and AOM/DSS induced colon cancer murine model, was shown to inhibit TH17 cell polarization and blocked colitis-related cancer (Lin et al.). Additionally, Zhang et al. reported that targeting miR-148b-5p in gastric cancer tumors reprogrammed the metabolic properties and altered the TME by shifting the lymphocyte and myeloid populations and rendered them sensitive to chemotherapeutic agents. These studies indicate modulation of tumor infiltrated immune cell properties and function through genetic and epigenetic mechanisms thereby affecting the TME.

## New Immunotherapeutic Opportunities With Pharmacological Inhibitors of Epigenetic Modifiers

The reversible nature of epigenetic marks has immense implications in the prevention and treatment of cancers. When combined with immunotherapeutic approaches, they provide an opportunity to design targeted therapies to affect a positive clinical outcome. Epigenetic modifiers have been tested in various preclinical and clinical studies with varying degrees of success. Saleh et al. provide a comprehensive review of epigenetic modifications on the regulation of immune checkpoint molecules, therapeutic approaches to epigenetic modifiers as therapy in clinical trials.

DNMT inhibitors, 5-azacytidine, and decitabine degrade and inhibit DNA methyltransferases, resulting in hypomethylation and re-expression of tumor suppressor genes such as CDKN2A in cancer cells (72, 73). DNMT inhibitors also increase the expression of PD-L1 and PD-L2 in melanoma. A similar effect was observed with class I HDAC inhibitors and, when combined with anti-PD1 therapy, suppressed the tumor progression and improved survival (74). As mentioned earlier, DNA methylation and repressive histone marks regulate the expression of immune checkpoint molecules in breast cancer and colorectal cancer (23, 75). In ovarian cancer cells, treatment with DNMT inhibitors through upregulation of previously hypermethylated endogenous retroviruses activated cytosolic sensing of double-stranded RNA, causing a type I interferon response and apoptosis (76). Combining DNMT inhibitor with HDAC6 inhibitor resulted in increased type I interferon response, leading to profound cytokine and chemokine expression and higher expression of the MHC I antigen presentation complex in human and mouse ovarian cancer cell lines (77). Approval of DNMT inhibitors for hematological malignancies has renewed interest in epigenetic therapy despite limited success in solid tumors (78, 79). A combination of low dose azacytidine and HDAC inhibitor entinostat in patients with non-small cell lung cancer resulted in durable responses and improved long-term survival (80).

Genetic abrogation or pharmacological inhibition of HDAC6 in melanoma cells decreased cell proliferation by inducing G1-cell cycle arrest without triggering apoptosis. This was also associated with increased expression of TAAs and MHC-1 molecules, indicating a greater role of HDAC6 in modulating anti-tumor immunity (81). HDAC6 is also reported to interact with STAT3 to regulate the expression of immunosuppressive cytokine, IL-10, by binding to the *IL10* gene promoter in antigen-presenting cells (82). Furthermore, inhibition of HDAC6 decreased STAT3 mediated expression of PD-L1 in primary melanoma samples and a panel of melanoma cell lines (83). Finally, using a murine melanoma model, pre-treatment with HDAC6 inhibitor prior to anti-PD1 immunotherapy resulted in decreased pro-tumor macrophages associated with increased infiltration of effector T-cells in the TME, providing evidence to the potential use of epigenetic modifiers as therapeutic agents for immunotherapy (84). In this Research Topic, similar results are presented in CLL whereby inhibition of HDAC6 augmented anti-PD1 and anti-PDL1 immunotherapy by increasing cytotoxic CD8 T-cell phenotype (Maharaj et al.). HDAC inhibitors also enhanced the expression of T-cell chemokines CXCL9 and CXCL10 and augmented anti-PD-1 immunotherapy response in lung adenocarcinoma (85).

## Future of Epigenetic Therapies

One of the downsides to using pharmacological approaches to inhibit or activate epigenetic modifiers is the lack of targeted effects, resulting in undesirable global changes that can discourage their usage as long-term cancer therapies. For example, treatment with HDAC inhibitors, despite having better toxicity profiles than traditional chemotherapeutic agents, caused patients’ suffering from gastrointestinal, hematological, and cardiac effects (86). Despite the limitations, epigenetic modifiers have been tested in the clinic in combination immunotherapeutic strategies. Several examples are as follows: BET inhibitor and atezolizumab (anti-PD-L1) combination is in a phase Ib open label trial in patients with advanced ovarian cancer or triple negative breast cancer (ClinicalTrials.gov Identifier: NCT0329217). Vorinostat which is an FDA-approved drug for cutaneous T-cell lymphoma is currently is under consideration to assess the early signals anti-tumor activity in combination with pembrolizumab (anti-PD1) in patients with advanced prostate, renal or urothelial cell carcinoma (ClinicalTrials.gov Identifier: NCT02619253). Phase I clinical trail to study the side effects and identify the best dose of class I HDAC inhibitor entinostat and nivolumab (anti-PD1) when given together with ipilimumab (anti-CTLA4) in treating patients with metastatic or unrescetable solid tumors that have spread to lymph nodes or other organ sites in human epidermal growth factor receptor 2 (HER2)-negative breast cancer patients (ClinicalTrials.gov Identifier: NCT02453620). In this Research Topic, Saleh et al. has provided an extensive list of latest clinical trials highlighting the increasing prominence of epigenetic drugs as immunomodulators of TME.

However, this issue can be addressed by developing highly selective isoform-specific HDAC inhibitors. Chronic, high doses of DNMT induce chromosomal instability and induce tumors in mice (87, 88). Therefore, a better approach would be to target a specific locus of chromatin to influence a desirable outcome. Targeted epigenetic modifications can be achieved by combining sequence-specific DNA binding domains with an epigenetic modifier. An early study showed that using synthetic zinc finger proteins fused to a library of about 223 yeast chromatin regulators can target specific locus (89).

Another example is an engineered transcriptional repressor fused to the catalytic domain of DNA methyltransferase (90). However, this in itself is a limiting factor as targeting different DNA sequences will require corresponding site-specific DNA binding domains. Other technologies include transcription activator-like effectors (TALEs) and RNA-guided clustered regularly interspaced short palindromic repeat (CRISPR) associated protein (Cas9) for precise epigenome editing (91, 92). With the advent of CRISPR technologies, we are entering a new frontier of targeted epigenomic therapies for cancer treatment.

## Author Contributions

SN wrote the manuscript and prepared the illustration. AV supervised and guided in preparing the manuscript. KC and LK reviewed and provided input and suggestions in writing the manuscript. All authors contributed to the article and approved the submitted version.

## Conflict of Interest

The authors declare that the research was conducted in the absence of any commercial or financial relationships that could be construed as a potential conflict of interest.

## Publisher’s Note

All claims expressed in this article are solely those of the authors and do not necessarily represent those of their affiliated organizations, or those of the publisher, the editors and the reviewers. Any product that may be evaluated in this article, or claim that may be made by its manufacturer, is not guaranteed or endorsed by the publisher.
